# Microbial diversity of thermophiles with biomass deconstruction potential in a foliage‐rich hot spring

**DOI:** 10.1002/mbo3.615

**Published:** 2018-03-30

**Authors:** Li Sin Lee, Kian Mau Goh, Chia Sing Chan, Geok Yuan Annie Tan, Wai‐Fong Yin, Chun Shiong Chong, Kok‐Gan Chan

**Affiliations:** ^1^ ISB (Genetics) Faculty of Science University of Malaysia Kuala Lumpur Malaysia; ^2^ Faculty of Biosciences and Medical Engineering Universiti Teknologi Malaysia Skudai Johor Malaysia; ^3^ Jiangsu University Zhenjiang China

**Keywords:** Biofilm, biofuel, biomass degradation, cellulase, hot spring, thermophile

## Abstract

The ability of thermophilic microorganisms and their enzymes to decompose biomass have attracted attention due to their quick reaction time, thermostability, and decreased risk of contamination. Exploitation of efficient thermostable glycoside hydrolases (GHs) could accelerate the industrialization of biofuels and biochemicals. However, the full spectrum of thermophiles and their enzymes that are important for biomass degradation at high temperatures have not yet been thoroughly studied. We examined a Malaysian Y‐shaped Sungai Klah hot spring located within a wooded area. The fallen foliage that formed a thick layer of biomass bed under the heated water of the Y‐shaped Sungai Klah hot spring was an ideal environment for the discovery and analysis of microbial biomass decay communities. We sequenced the hypervariable regions of bacterial and archaeal 16S rRNA genes using total community DNA extracted from the hot spring. Data suggested that 25 phyla, 58 classes, 110 orders, 171 families, and 328 genera inhabited this hot spring. Among the detected genera, members of *Acidimicrobium*,* Aeropyrum*,* Caldilinea*,* Caldisphaera*,* Chloracidobacterium*,* Chloroflexus*,* Desulfurobacterium*,* Fervidobacterium*,* Geobacillus*,* Meiothermus*,* Melioribacter*,* Methanothermococcus, Methanotorris*,* Roseiflexus*,* Thermoanaerobacter*,* Thermoanaerobacterium*,* Thermoanaerobaculum*, and *Thermosipho* were the main thermophiles containing various GHs that play an important role in cellulose and hemicellulose breakdown. Collectively, the results suggest that the microbial community in this hot spring represents a good source for isolating efficient biomass degrading thermophiles and thermozymes.

## 1 INTRODUCTION

Lignocellulolytic biomass is a sustainable resource for second‐generation biofuel production (Xia, Ju, Fang, & Zhang, 2013). Global demand for biofuel, combined with the depletion of nonrenewable fossil fuels, has resulted in the rapid expansion of biofuel production (Xing, Zhang, & Huang, 2012). Fungi and bacteria are major decomposers of organic matter, with bacteria being more influential due to their metabolic versatility (Liu et al., 2011; López‐González et al., 2015). Although fungi have been widely used for lignocellulolytic biomass degradation, bacteria have an equally important role in the degradation process (Takaku, Kodaira, Kimoto, Nashimoto, & Takagi, 2006; Watanabe, Nagao, Toda, & Kurosawa, 2009; Partanen, Hultman, Paulin, Auvinen, & Romantschuk, 2010; Karadag et al., 2013). Bacteria also have a remarkable ability to tolerate changes in environmental conditions compared to fungi, including extreme pH and temperature (Li et al., 2013). Research has been focusing on finding new thermostable enzymes or isolate thermophilic cells for lignocellulolytic degradation. The bacteria class Clostridia and its order *Thermoanaerobacterales* have been extensively studied (Chang & Yao, 2011). Additionally, *Deinoccocus‐Thermus* spp. (Wu et al., 2015), *Geobacillus* spp. (Zambare, Bhalla, Muthukumarappan, Sani, & Christopher, 2011; Brumm, et al., 2015; Brumm, Land, & Mead, 2015), *Melioribacter* spp. (Rakitin, Ermakova, & Ravin, 2015), and *Thermoanaerobacterium* spp. (Currie et al., 2014) are known for decomposing biomass at high temperatures (Bhalla, Bansal, Kumar, Bischoff, & Sani, 2013). Furthermore, members of the genera *Thermotoga* (Yu et al., 2016), *Rhodothermus* (Keshk, 2016), *Anoxybacillus* (Chan et al., 2016), *Rhodothermaceae* strain RA (Goh et al., 2016), and *Caldicellulosiruptor* (Peng et al., 2015) were found to produce thermostable enzymes for biomass saccharification.

Thermostable biomass‐acting enzymes are promising due to their suitability for industrial applications (Duan & Feng, 2010). Other advantages of thermophiles and their enzymes have been reported (Taylor et al., 2009; Vishnivetskaya et al., 2015). Heated environments such as hot springs are potential sources of thermophiles and thermozymes (Urbieta et al., 2015; Zhao et al., 2017). Due to high temperatures, most known hot springs lack vegetation sources. In one report, the microbial community in heated (68°C) sediments surrounding vegetated (*Juncus tweedyi*) wetland in Obsidian Pool (site OBP10) in Yellowstone National Park (YNP) was found to mainly include *Firmicutes*,* Proteobacteria*,* Aquificae*,* Deinococcus‐Thermus*,* Spirochaetes*, and *Verrucomicrobia* phyla, and a huge proportion of unclassified bacteria. The majority of *Firmicutes* members including lignocellulolytic degraders were *Clostridium*,* Anaerobacter*,* Caloramator*,* Caldicellulosiruptor*, and *Thermoanaerobacter* (Vishnivetskaya et al., 2015). When OBP10 samples were inoculated with various lignocellulolytic materials, including Avicel, switchgrass, *Populus*, and xylan, and incubated at 55–85°C in anaerobic laboratory conditions, the main bacteria after three culturing rounds were *Thermoanaerobacter*,* Caloramator*,* Caldicellulosiruptor*,* Clostridium*,* Dictyoglomus*, and *Fervidobacterium*; their distributions in these experiments varied with experimental parameters such as temperature and type of substrate (Vishnivetskaya et al., 2015).

Another site that lacks lignocellulosic plant material is the Great Boiling Spring (GBS), located in Nevada (77–85°C) (Peacock et al., 2013). Microbial diversity analysis was conducted to compare the microbial diversity in GBS water‐sediments with man‐made in situ enrichment using ammonia fiber explosion‐treated corn stover and aspen shavings. The microbial community attached to the supplemented biomass consisting of potential biomass degraders, sugar fermenters, and hydrogenotrophs that included *Thermotoga*,* Dictyoglomus*,* Desulfurococcales*, and *Archaeoglobales*. The microbial flora in biomass‐enriched samples and GBS indigenous samples were different. Therefore, Peacock et al. (2013) suggested that the additional lignocellulosic biomass stimulated the growth of the potent biomass degraders in a natural environment.

One of the quickest approaches for examining microbial populations is 16S rRNA amplicon sequencing. The genera involved in high‐temperature biomass degradation have been studied in laboratory setups with a predefined medium or type of biomass (Park et al., 2012; Eichorst et al., 2013; Peacock et al., 2013; Xia et al., 2014; Vishnivetskaya et al., 2015; Yu et al., 2015). In this study, we analyzed the microbial diversity in a Malaysian hot spring (60–90°C, mean 68°C, pH 8.6) using 16S rRNA amplicon‐based sequencing. The Y‐shaped Sungai Klah (SK‐Y) hot spring was studied because it is a natural “biomass degrading bioreactor” due to the presence of a submerged foliage bed. The data and results obtained add to the list of important thermophiles for biomass degradation at high temperatures, suggesting that the microbial populations involved in biomass degradation in natural environments are far more complicated than in laboratory setups.

## 2 MATERIALS AND METHODS

### 2.1 Sample collection and water analysis

The Y‐shaped Sungai Klah hot spring (SK‐Y) (3°59′50.50″N, 101°23′35.51″E) is located in Perak, Malaysia. Previously, we conducted microbial diversity analysis of the main water source of the Sungai Klah (SK) hot spring (Chan, Chan, Tay, Chua, & Goh, 2015). In this work, samples of the trapped heated spring water were taken from the SK‐Y. The SK‐Y is located approximately 10 meters from the SK hot spring, as reported by Chan et al. (2015).

Sampling was performed on March 24, 2016. A clean stainless water sampling dipper was used to collect water samples without any foliage at four different spots with approximately 5 m between sampling locations. Water was stored in sterile glass Schott bottles and immediately transported to the laboratory within 2.5 hr and stored at 4°C overnight. On the following day, water analysis was conducted by MyTest Lab Sdn Bhd (Malaysia) using American Public Health Association standard protocols. At least 20 pieces of submerged foliage with no apparent biofilm were collected with a sampling dipper and transferred to polypropylene Ziploc bags using a tweezer. Submerged foliage with green biofilm was collected and stored separately in 1 L Schott bottles. Degraded foliage was collected at the base of SK‐Y with green biofilm, and nondegraded plant litters were carefully removed in situ.

Freshly picked foliage samples from trees growing along the SK‐Y were collected and stored in sterile polyethylene bags. The lignin, cellulose, and hemicellulose content of these foliage samples were analyzed at the Malaysian Agricultural Research and Development Institute (MARDI), a local service provider of acid detergent lignin (ADL), acid detergent fiber (ADF), and neutral detergent fiber (NDF) analysis using a modified protocol based on Van Soest and Wine (1967).

### 2.2 Total community DNA extraction

Unless specified, all samples were kept at 4°C. DNA extraction was completed within 2 days after sample collection. To study the microbial diversity of the SK‐Y, total community DNA extraction of the following samples was performed: (1) pooled water of four sites with an equal volume ratio, (2) submerged foliage with no apparent biofilm, (3) submerged foliage with green biofilm, and (4) degraded foliage collected at the base of the SK‐Y.

Four liters of pooled water were filtered through a filter membrane with a 0.22‐μm pore size (Sartorius, Göettingen, Germany). Then, the membrane was placed in 10 ml of autoclaved 1 ×  concentration phosphate‐buffered saline (PBS; 137 mmol/L sodium chloride, 2.7 mmol/L potassium chloride, and 10 mmol/L phosphate buffer) containing sterile glass beads and shaken vigorously for 5 min. Next, the membrane was removed and the leftover liquid was centrifuged at 17,000*g* for 2 min at 4°C. The supernatant was discarded and the pellet was resuspended using Tris‐EDTA buffer (10 mmol/L Tris‐HCl (pH 7.5), 1 mmol/L EDTA) and modified cetyltrimethylammonium bromide (CTAB) lysis buffer (100 mmol/L Tris‐HCl, 100 mmol/L EDTA, 100 mmol/L K_2_HPO_4_, 1.5 mol/L NaCl, and 1% w/v CTAB) (Murray & Thompson, 1980; Zhou, Bruns, & Tiedje, 1996). Subsequently, enzymatic lysis was performed with overnight incubation at 37°C in the presence of 10 mg/ml of lysozyme (Sigma‐Aldrich, Saint Louis, MO, U.S.) and gently swirled at 20‐min intervals. The solution was then incubated at 90°C for 1 hr (Murray & Thompson, 1980). Subsequently, sodium dodecyl sulfate was added to the final concentration of 1% (w/v), followed by the introduction of 20 mg/ml of proteinase K (Qiagen, Valencia, CA, U.S.), incubated at 60°C for 2 hr with gentle shaking at 15‐min intervals. RNA was broken down using 100 mg/ml RNase A (Qiagen) and incubated at 37°C for 30 min. Proteins were removed by washing the DNA pellet with phenol/chloroform/isoamyl alcohol (25:24:1). The resulting DNA pellet was precipitated with 0.6 volume of isopropanol, followed by 70% (v/v) ethanol, and was rehydrated with 60 μl of elution buffer (Qiagen) (Manjula, Sathyavathi, Gunasekaran, & Rajendhran, 2011).

Approximately 100 g of foliage samples were separately placed according to sample type (foliage with no apparent biofilm, foliage with green biofilm, or degraded foliage) inside a 500 ml autoclaved glass bottle containing sterile PBS with 0.05% Tween 20 (4 ml PBS per 1 g leaf, pH 7.4), and sonicated (Branson Ultrasonics, Danbury, CT, U.S.) for 1 min at 25°C. The preparations were then hand‐shaken vigorously for 30 s, and leaf debris was discarded, and the remaining liquid was aliquoted into a 50 ml tube and centrifuged at 14,800*g* for 10 min at 4°C. The pellet was subjected to the aforementioned conventional total community DNA extraction.

To improve the purity of the extracted total community DNA, inhibitors such as humic acids were removed using the Agencourt AMPure XP System (Beckman Coulter, Brea, CA, U.S.). Quality and yield of the purified total community DNA were examined using 1% w/v agarose gel electrophoresis, a Nanodrop^™^ 1000 spectrophotometer (Thermo Scientific, Waltham, MA, U.S.), and a Qubit^®^ 2.0 Fluorometer (Invitrogen, Merelbeke, Belgium).

### 2.3 Library construction and 16S rRNA amplicon‐based sequencing

The Illumina 16S rRNA Metagenomic Sequencing Library Preparation Guide was followed for the preparation of the libraries. Two sets of primers were used to target bacterial and archaeal hypervariable 16S rRNA conserved regions (Klindworth et al., 2012): (1) 16S rRNA V3 and V4 bacterial amplicon polymerase chain reaction (PCR) forward primer 5′‐TCG TCG GCA GCG TCA GAT GTG TAT AAG AGA CAG CCT ACG GGN GGC WGC AG‐3′; (2) 16S rRNA V3 and V4 bacterial amplicon PCR reverse primer 5′‐GTC TCG TGG GCT CGG AGA TGT GTA TAA GAG ACA GGA CTA CHV GGG TAT CTA ATC C‐3′; (3) targeted archaeal amplicon PCR forward primer 5′‐TCG TCG GCA GCG TCA GAT GTG TAT AAG AGA CAG CMG CCG CGG TAA‐3′; and (4) targeted archaeal amplicon PCR reverse primer 5′‐GTC TCG TGG GCT CGG AGA TGT GTA TAA GAG ACA GTA CNV GGG TAT CTA ATC C‐3′. The underlined and nonunderlined sequences refer to the Illumina adapter overhang nucleotide sequences and locus‐specific primers for the regions to be targeted, respectively. The amplicons were then subjected to a series of library quantification steps to accurately quantify the NGS (next‐generation sequencing) sample libraries. The amplicons were quantified with Qubit dsDNA HS Assay Kit (Invitrogen) on a Qubit^®^ 2.0 Fluorometer, and the library size was selected based on Agilent Technologies 2100 Expert Bioanalyzer using Agilent High Sensitivity Kit (Agilent Technologies, Santa Clara, CA, U.S.). The number of amplifiable molecules in a library were quantified absolutely using Eco Real‐Time PCR System with KAPA Library Quantification Kit (KAPA BioSystems, Boston, MA, U.S.) prior to sequencing. Next, libraries were sequenced using paired‐end sequencing on an Illumina MiSeq sequencer (Illumina, San Diego, CA, U.S.) with MiSeq Reagent Kit V2 (2 × 250 base pairs) and V3 (2 × 300 base pairs) for the archaeal primers amplicons and bacterial primers amplicons, respectively.

### 2.4 Sequence analysis

The raw sequence reads generated by the Illumina sequencer were processed in CLC Genomic Workbench 7.0 (CLC Bio, Aarhus, Denmark). Adapter sequences were trimmed and reads were filtered to ensure an average Phred score of 20. Paired‐end reads were merged (mismatch cost = 2; gap cost = 3; maximum unaligned end mismatches = 0; minimum score = 8) in CLC Genomics Workbench 7.0. The assembled reads were then subjected to chimera filtering and microbial taxonomic classification using the Quantitative Insights Into Microbial Ecology (QIIME) pipeline (Caporaso et al., 2010). Analyses performed using the QIIME (version 1.9.1) pipeline were based on default parameters, unless otherwise stated. Briefly, the steps included removing chimera sequences, picking operational taxonomic units (OTUs) based on an open reference clustering approach using the UCLUST tool, and taxonomic assignment using BLAST with the National Center for Biotechnology Information (NCBI) 16S Microbial database with an e‐value of 0.001. The NCBI database was selected because it is large and diverse, with the capability to provide a greater depth of information during taxonomic profiling compared to RDP, GreenGenes, or SILVA databases (Chan et al., 2015). All samples were randomly subsampled to the same sequencing depth prior to analysis. Microbial diversity was assessed using rarefraction analysis, the number of observed OTUs per sample, Shannon–Wiener, and Simpson using QIIME. Beta diversity measurements between all the samples were calculated using Unifrac distance (Lozupone & Knight, 2005), implemented in QIIME. Principal coordinates analysis (PCoA) was performed on the weighted UniFrac distance matrix, which accounts for communities’ membership and relative abundance of OTUs. The resulting sequencing data were submitted to NCBI SRA under Bioproject PRJNA353967.

### 2.5 Carbohydrate‐active gene prediction

After taxonomic assignment using QIIME, taxa with a relative abundance of ≥0.85% were individually checked against the complete genome information available in the Carbohydrate‐Active EnZymes database (CAZy) to determine the number and types of GH families for these taxa.

## 3 RESULTS

### 3.1 General site descriptions

**Figure 1:**
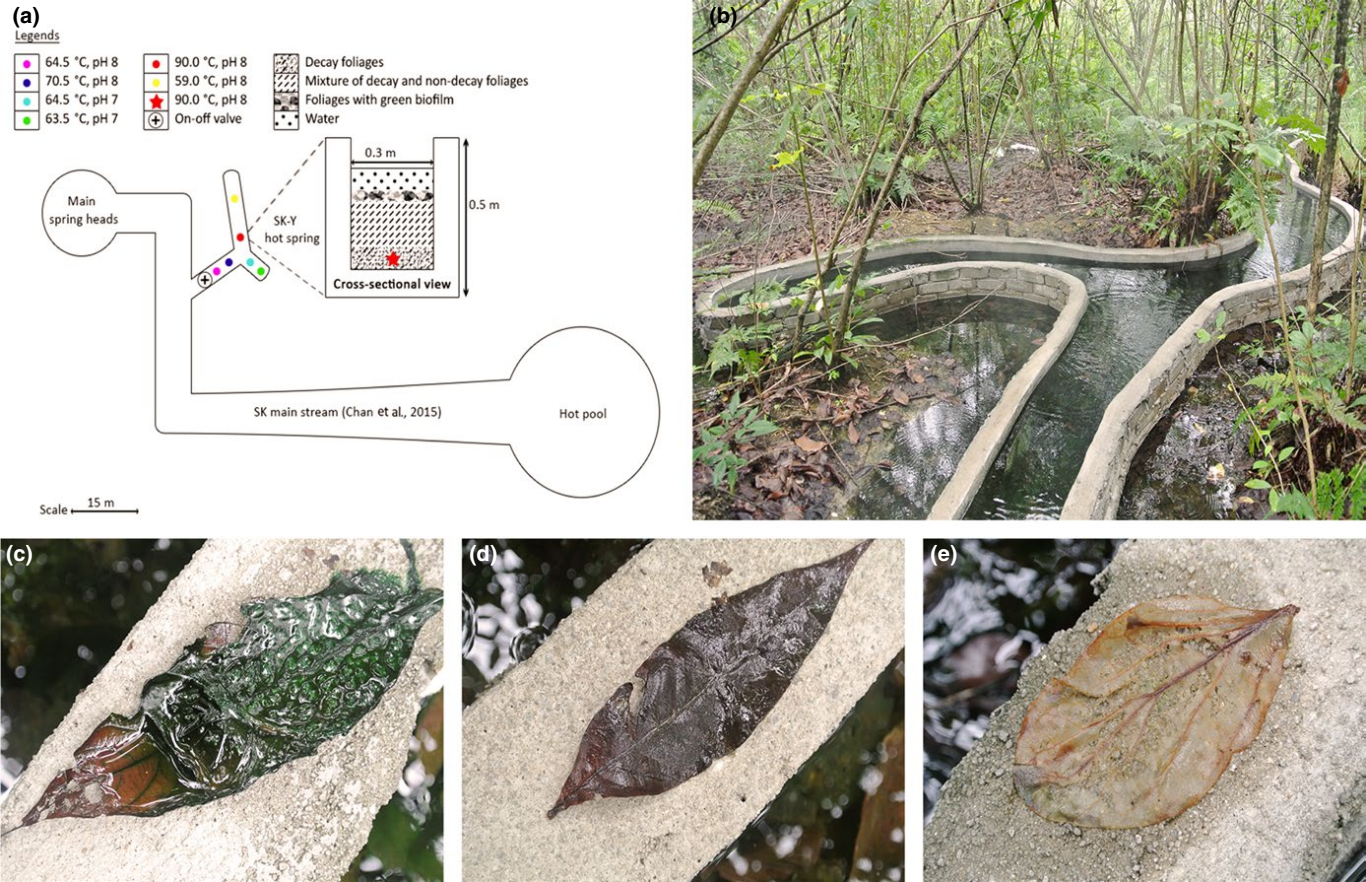
Y‐shaped Sungai Klah hot spring (SK‐Y) and types of samples. (a) Illustration of sampling site for water and foliage, (b) SK‐Y, (c) foliage with green biofilm, (d) nondegraded foliage with no apparent biofilm, and (e) degraded foliage

More than a dozen hot spring sites are present in the Sungai Klah hot spring park. Previously, we performed a 16S rRNA amplicon and shotgun sequencing for samples obtained from one of the SK hot springs (Chan et al., 2015). Although the site is located in a wooded area, plant litter does not accumulate in the hot spring, as the stream flows rapidly. Approximately 10 m away from the previously studied location (Chan et al., 2015), man‐made drainage, 30 m long and 0.5 m deep, was built to trap heated spring water (Figure 1a). As the shape of the drainage is Y‐shaped, we therefore named the samples obtained from this site as SK‐Y, to differentiate current work from our earlier study that was identified as SK hot spring (Chan et al., 2015). The temperature at the SK‐Y spring head was approximately 90°C, but was lower (60–70°C; mean 68°C) adjacent to the water's surface and further away from the spring head. The pH for SK‐Y ranged between 7.5 and 8.6. The most interesting feature of SK‐Y is the presence of fallen plant litter that is mainly foliage, accumulated in the heated water, resulting in the formation of a bed of foliage with a thickness of approximately 20 cm. The average size of the fresh fallen foliage is about 15 cm long by 6 cm wide.

### 3.2 Physicochemical analysis of water

Water analysis was completed to determine the physicochemical condition of SK‐Y (Table S1). The temperature and pH during sampling were 68°C and 8.6, respectively. The color of the SK‐Y water was 68 true color units (TCU). Aluminum (0.96 mg/L) and iron (0.65 mg/L) were detected in the SK hot spring (Chan et al., 2015), but these metal ions were not detected in the SK‐Y water sample. SK‐Y has higher fluoride (6 mg/L), nitrate (0.29 mg/L), and zinc (0.17 mg/L) content compared to the SK hot spring (1.1 mg/L, <0.1 mg/L, and <0.02 mg/L, respectively). The sulfur and sulfide content in SK‐Y were 0.5 mg/L and 12.3 mg/L, respectively. In SK‐Y, other metals such as mercury, cadmium, chromium, lead, manganese, nickel, silver, aluminum, barium, and strontium were below quantifiable limits.

### 3.3 Analysis of foliage lignocellulose content

*Vitex*,* Ficus*,* Stenochlaena*, and *Adenanthera* are the main plant genera that grew adjacent to SK‐Y. Most of these plants are approximately 2–4 m in height. The average percentage of lignin, cellulose, and hemicellulose for foliage randomly picked from the trees were determined using NDF (hemicellulose, cellulose, and lignin), ADF (cellulose and lignin), and ADL (fractions of plant cell walls) (Van Soest & Wine, 1967). The lignin, cellulose, and hemicellulose contents of the foliage are shown in Table 1. The lignin content of the foliage samples varied from 3.0% to 16.7%, whereas hemicellulose content ranged between 2.9 and 4.5%. *Vitex* and *Stenochlaena* samples were relatively higher in cellulose content than the *Ficus* and *Adenanthera* samples. The lignocellulosic content of the plant genera (*Ficus*,* Stenochlaena*, and *Adenanthera*) were not found in the literatures to date, except as reported by Codron, Lee‐Thorp, Sponheimer, and Codron (2007) for *Vitex* sp. foliage, which stated that foliage contained 6.0% lignin, 7.2% cellulose, and 8.1% hemicellulose.

**Table 1:** Approximate composition (as a percentage) of various foliage samples

Genus	Lignin (%)	Hemicellulose (%)	Cellulose (%)
*Vitex*	9.9	3.9	10.2
*Ficus*	8.7	2.9	7.0
*Stenochlaena*	16.7	4.3	11.3
*Adenanthera*	3.0	4.5	3.8

### 3.4 16S rRNA gene sequencing data analysis

Total community DNA were extracted for four different types of samples. These sample types were (1) SK‐Y water, (2) submerged foliage with no apparent biofilm (labeled as nondecay), (3) submerged foliage with green biofilm (labeled as green biofilm), and (4) degraded foliage (labeled as decay) (Table 2). The top layer of the submerged plant litter bed is always covered with green biofilm (soft in texture) (Figure 1c). Foliage decomposes underneath at the base of the bed (Figure 1e).

**Table 2:** Summary of assembled data obtained from total community DNA of water and foliage microbiota

Dataset		SK‐Y water		Green biofilm		Nondecay		Decay	
		Bacteria	Archaea	Bacteria	Archaea	Bacteria	Archaea	Bacteria	Archaea
Number of reads		510983	1153627	248958	1455515	288476	1437865	651444	826285
Sequence length (bp)	Minimum	200	200	264	203	264	200	200	200
	Average	294	266	269	263	269	263	329	261
	Maximum	429	422	275	390	275	390	430	320
Observed OTUs		11704	31	16331	26	16153	21	15529	21
Shannon		7.867	2.195	7.450	0.961	7.318	0.574	7.169	1.402
Simpson		0.989	0.533	0.982	0.242	0.982	0.141	0.981	0.405

Total community DNA extracts for water, nondecay, green biofilm, and decay underwent 16S rRNA amplicon‐based sequencing using primer pairs specific for bacteria and archaea. After quality filtration and adapter trimming of the raw reads, high‐quality assembled reads were analyzed using QIIME pipeline (Table 2). An average of 14,929 and 25 observed OTUs for bacteria and archaea were generated from four samples, respectively. These data were processed at a rarefaction depth of 819,322 and 12,175 sequences per bacteria and archaea sample, respectively. Simpson and Shannon–Wiener indexes indicated the highest species richness and evenness in the bacterial diversity of the SK‐Y water sample, whereas the lowest was found in the archaeal diversity of nondecay sample. The rarefaction curves of all the samples did not reach saturation, indicating that there is high level of diversity in the systems (Figures S1A and S1B).

To assess the microbial phylogenetic beta diversity, we used the weighted Unifrac distance, which indicates the extent of the phylogenetic similarities among the microbial communities (Lozupone, Hamady, Kelley, & Knight, 2007). PCoA using weighted UniFrac revealed that the bacterial communities (Figure S2A) of the samples were grouped into three distinct clusters. Overall, bacterial communities were phylogenetically more similar between the green biofilm and nondecay samples, whereas a clear difference was revealed between the SK‐Y water, decay, and cluster of green biofilm and the nondecay samples. The same pattern was also found in archaeal communities (Figure S2B).

### 3.5 Bacterial diversity analysis

**Figure 2:**
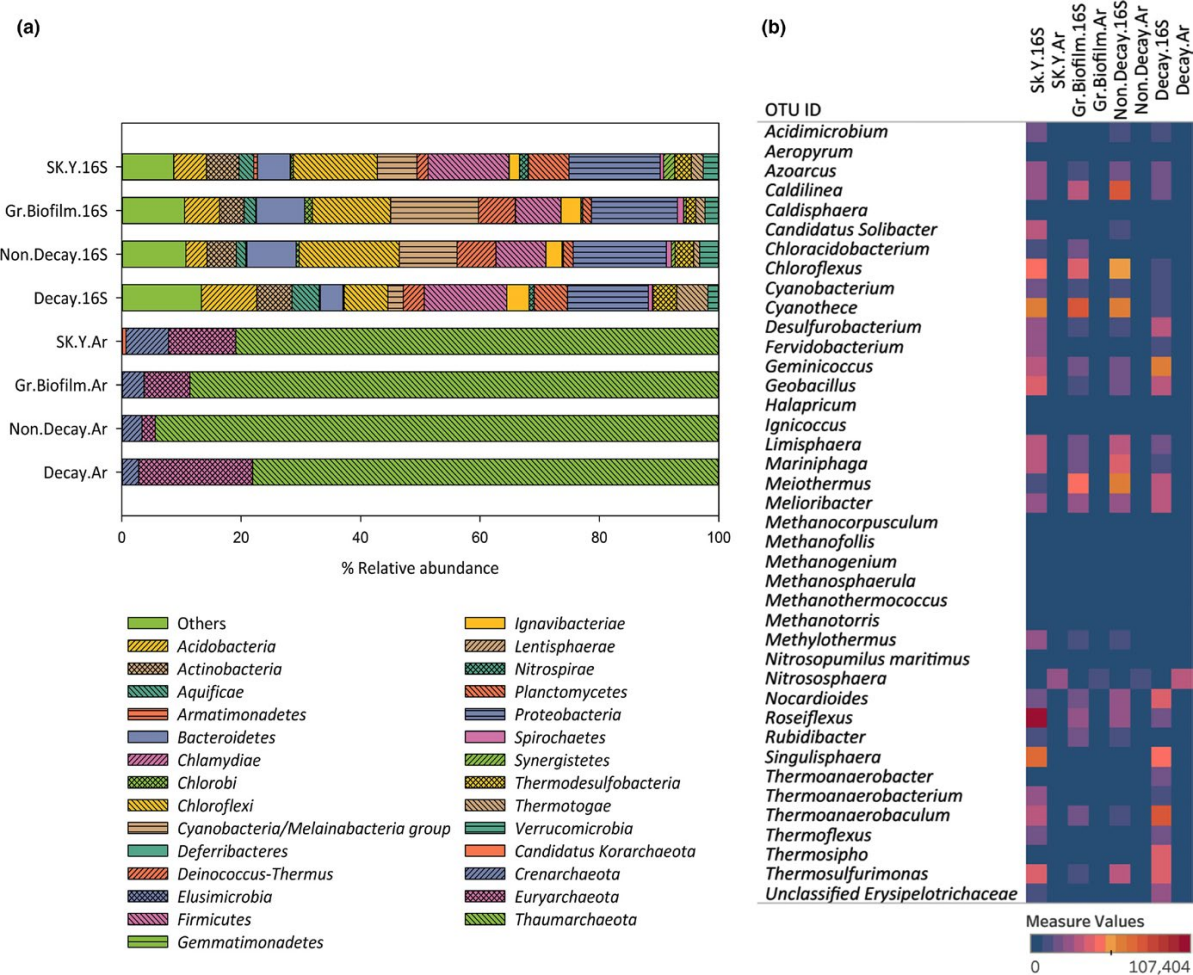
(a) Relative abundance at bacterial and archaeal phyla in SK‐Y and (b) heat map representation of abundance bacterial (≥ 0.85%) and archaeal genera in SK‐Y

Unless specified, the values shown in this subsection are the average number for the four samples. Generally, the taxonomic assignment of bacteria in the four samples could be classified into 25 phyla, 58 classes, 110 orders, 171 families, and 328 genera. The 10 most abundant phyla contributed 75.5% of the total bacterial diversity, including *Proteobacteria* (14.7%), *Chloroflexi* (12.8%), *Firmicutes* (10.8%), and *Cyanobacteria* (8.4%) (Figure 2a). The minor phyla present in the SK‐Y samples included *Chlamydiae*,* Gemmatimonadetes*,* Elusimicrobia*,* Lentisphaerae*, and *Deferribacteres*, which were each ≤0.08% of the total population. OTUs affiliated with *Armatimonadetes* (0.28%), *Chlorobi* (0.60%), *Nitrospirae* (0.70%), *Spirochaetes* (0.78%), and *Synergistetes* (0.78%) were also detected at lower percentages. The decay sample had higher percentages of *Acidobacteria*,* Aquificae*, and *Thermotogae* compared to the other three samples. Photosynthetic phyla, such as *Chloroflexi* and *Cyanobacteria*, were present in lower percentages in the decay sample than those in green biofilm. A broad range of *Proteobacteria*, including *Alpha*‐, *Beta*‐, *Gamma*‐, *Delta*‐, and *Epsilonproteobacteria*, was observed in all samples.

The following classes contributed to almost 69% of the total population in the degraded foliage microbiota: *Actinobacteria*,* Aquificae*,* Deinococci*,* Bacilli*,* Clostridia*,* Ignavibacteria*,* Planctomycetia*,* Alphaproteobacteria*,* Thermodesulfobacteria*,* Thermotogae*, unclassified *Acidobacteria*, and other unclassified classes or blast hits. All these classes were also present in green biofilm, nondecay, and water samples, but occurred in different proportions. The main classes associated with green biofilm, but present in lower percentages in the decay sample, included *Blastocatellia*,* Bacteroidia*,* Cytophagia*,* Caldilineae*,* Chloroflexia*,* Cyanobacteria*,* Betaproteobacteria*,* Deltaproteobacteria*, and *Gammaproteobacteria*.

A total of 171 families were observed across all the samples, with *Thermaceae* (4.5%), *Caldilineaceae* (3.8%), *Chloroflexaceae* (3.6%), and *Roseiflexaceae* (3.6%) representing the most abundant groups. In addition, *Fervidobacteriaceae* (5.0%) was found to be the predominant family in the decay sample. For bacterial genera, the four samples in SK‐Y shared most OTUs but with different abundances (Figure 2b), dominated by *Caldilinea* (3.8%), *Meiothermus* (3.8%), *Chloroflexus* (3.6%), *Roseiflexus* (3.6%), *Thermoanaerobaculum* (3.1%), *Melioribacter* (2.4%), *Geobacillus* (2.2%), *Desulfurobacterium* (1.8%), *Thermosipho* (1.3%), *Thermoanaerobacterium* (1.0%), *Fervidobacterium* (1.0%), *Acidimicrobium* (1.0%), *Chloracidobacterium* (1.0%), and *Thermoanaerobacter* (0.9%).

### 3.6 Archaeal diversity analysis

In addition to bacteria, we wanted to understand the involvement of archaea in biomass degradation. An archaeal specific primer pair was used to target locus‐specific sequences of archaea in four of the samples taken from SK‐Y (Figure 2a). Although the primers were specifically designed for archaea, only 0.9%–3.8% of the OTUs identified in these four samples were affiliated with archaea. From this, we hypothesized that archaea are present in a relatively small portion in SK‐Y. Further analysis using shotgun metagenome sequencing was needed to validate this assumption. For subsequent analysis of archaea, all OTUs related to bacteria were excluded. The most prevalent phylum of archaeal diversity across all samples was *Thaumarchaeota* (85.5%). In SK‐Y water, green biofilm, and decay samples, *Euryarchaeota* was the second‐most abundant archaeal phylum, followed by *Crenarchaeote*. *Crenarchaeota* was the second‐most dominant phylum in the nondecay sample, followed by *Euryarchaeota*. Within *Thaumarchaeota*, the most abundant class in all samples was *Nitrososphaeria*, with *Nitrososphaera* (84.0%) as the dominant genus. Furthermore, the next most abundant class in SK‐Y water, green biofilm, and decay samples was *Methanomicrobia*, mainly represented by *Methanofollis*. *Thermoprotei* was the second‐most dominant class in nondecay sample with *Aeropyrum* as the second abundant genus. Genus level assignment revealed that *Methanocorpusculum* (3.6%), *Nitrosopumilus* (1.5%), *Methanogenium* (0.4%), *Ignicoccus* (0.3%), *Methanotorris* (0.3%), *Methanosphaerula* (0.3%), *Halapricum* (0.2%), and *Methanothermococcus* (0.2%) genera were present in all the samples (Figure 2b). In contrast, *Caldisphaera* genus was found in all samples except for the nondecay sample.

### 3.7 Thermophiles and thermozymes involved in foliage degradation

The majority of detected OTUs were confidently assigned to the genus level with a blast e‐value of 0.001. Genera with a relative abundance of ≥0.85% in at least one SK‐Y sample were shortlisted and searched for related literature and databases. Table S2 summarizes the representative strains of these genera that were previously sequenced with complete genome information. We found that the following thermophilic bacterial genera have an abundance of genes encoded for 61 GH sequences: *Acidimicrobium*,* Caldilinea*,* Chloracidobacterium*,* Chloroflexus*,* Desulfurobacterium*,* Fervidobacterium*,* Geobacillus*,* Meiothermus*,* Melioribacter*,* Roseiflexus*,* Thermoanaerobacter*,* Thermoanaerobacterium*,* Thermoanaerobaculum*, and *Thermosipho*. For archaeal genera, *Aeropyrum*,* Caldisphaera*,* Methanotorris*, and *Methanothermococcus* are potent lignocellulosic biomass degraders, yet the total number of GHs for these archaeal genera were relatively few compared to bacterial genera (Table S2). A comparison of the major biomass degraders found in this study and selected literature is shown in Table 3.

**Table 3:** Comparison of the potential thermophilic biomass degraders in different experimental setups

Source	Potential biomass degraders	Analysis approaches	Temp., (°C)	pH	Biomass substrates/source	References
Submerged foliage and hot spring water of SK‐Y, Perak, Malaysia	*Acidimicrobium, Aeropyrum, Caldilinea, Caldisphaera, Chloracidobacterium, Chloroflexus, Desulfurobacterium, Fervidobacterium, Geobacillus, Meiothermus, Melioribacter, Methanothermococcus, Methanotorris, Roseiflexus, Thermoanaerobacter, Thermoanaerobacterium, Thermoanaerobaculum, Thermosipho*	Cultivation‐independent	60–70	7.5–8.6	Plant litter	This study
Sediments from hot spring, Xiamen, China	*Geobacillus*,* Thermus*,* Bacillus*,* Anoxybacillus*	Enrichment	50–80	7.0	Sugarcane bagasse	Zhao et al. (2017)
Mixture of water and sediment from SK main stream hot spring, Perak, Malaysia	*Aciduliprofundum, Caloramator, Hydrogenobacter, Ignavibacterium, Melioribacter, Methanocaldococcus, Methanocella, Methanothermus, Methylacidiphilum, Thermodesulfovibrio, Thermotoga, Thermus*	Cultivation‐independent	50–110	7.0–9.0	Scattered plant litter	Chan et al. (2015)
Soil contacting regions of a bagasse pile at Phu Khieo Bio‐Energy Chaiyaphum province, Thailand	*Actinobacteria, Bacteroidetes/Chlorobi, Chlamydiae*/*Verrucomicrobia, Chloroflexi, Fibrobacteres*/*Acidobacteria, Firmicutes, Planctomycetes, Proteobacteria*	Cultivation‐independent	50	n.a	Sugarcane bagasse	Mhuantong et al. (2015)
Vegetated area of Obsidian Pool (site OBP 10), Yellowstone National Park	*Anaerobacter, Caldicellulosiruptor, Caloramator, Clostridium, Thermoanaerobacter*	Cultivation‐independent and enrichment	55–85	5	In situ sampling (*Juncus tweedyi*); enrichment (Avicel, xylan, switchgrass, *Populus*)	Vishnivetskaya et al. (2015)
Anaerobic digestion sludge collected from Shek Wu Hui wastewater treatment plant, Hong Kong, China	*Anaerolineales, Bacteroidales, Clostridiales, Methanobacteriales, Methanosarcinales, Thermotogales*	Enrichment	55	6.0–7.0	Microcrystalline cellulose with glucose	Xia et al. (2014)
Sediment and water column of Great Boiling Spring, Nevada	*Archaeoglobales*,* Desulfurococcales, Dictyoglomus, Thermotoga*	*In situ* enrichment	74–85	5	Ammonia fiber explosion‐treated corn stover and aspen shavings	Peacock et al. (2013)
Switchgrass‐adapted bacterial consortia	*Paenibacilli* spp.*, Rhodothermus marinus, Thermobispora bispora, Thermomicrobia* sp., *Thermus thermophilus*	Enrichment	60	n.a	Microcrystalline cellulose	Park et al. (2012)

## 4 DISCUSSION

### 4.1 Microbial diversity analysis in SK‐Y

To analyze microbial diversity, 16S rRNA amplicon‐based sequencing has been widely used in various engineered or environmental samples (Hess et al., 2011; Singh et al., 2014; Chan et al., 2015; Mhuantong, Charoensawan, Kanokratana, Tangphatsornruang, & Champreda, 2015; Vishnivetskaya et al., 2015). Culture independent studies at hot springs in YNP have a long research history where the clonal library was initially used (Brock, 1967; Thiel et al., 2016). Microbial diversity in Octopus and Mushroom Springs at YNP were revisited using high‐throughput NGS (Thiel et al., 2016). Most of the studied hot springs lack lignocellulosic plant materials.

Although SK (Chan et al., 2015) and SK‐Y hot springs are approximately 10 m apart, the dominant microbial diversity was found to be dissimilar, probably due to several factors such as physicochemical or geochemical structure, temperature, dissolved oxygen level, and the quantity of plant litter. It is known that abiotic factors collectively contribute to the dynamics of microbial populations (Chan et al., 2017). In comparison to some known acidic hot springs (Lombard, Ramulu, Drula, Coutinho, & Henrissat, 2014; Sharp et al., 2014), SK‐Y demonstrated rich microbial diversity. Often, microbial diversity might be higher in circumneutral or slightly alkaline hot springs than those of acidic sites (Sharp et al., 2014).

SK‐Y was studied in this work because it represents a natural biomass degrading bioreactor. The top surface of the submerged foliage bed was covered by a green biofilm. Microbial diversity analysis showed that *Cyanobacteria* (14.7%), *Proteobacteria* (14.4%), and *Chloroflexi* (13.1%) were the main three phyla that contributed to green biofilm communities. *Cyanobacteria* and *Chloroflexi* are chlorophyll‐based phototrophic bacteria. Their growth rates are strongly affected by temperature, pH, sulfide concentration, sunlight, and other factors (Klatt et al., 2013). A significant amount of sulfide (12.3 mg/L) with different chemical compositions were present in SK‐Y (Table S1), which could potentially lead to increases in the abundance of chlorophototrophic bacteria in SK‐Y. Moreover, the stagnant spring water of SK‐Y has favored the green biofilm formation, compared to our previously studied fast‐flowing SK hot spring (sulfide, 0.2 mg/L) with a lower detected abundance of chlorophototrophs (Chan et al., 2015).

This study shows that *Firmicutes* and *Proteobacteria* were the dominant phyla in the microbiota of degraded foliage in the deeper part of the hot spring. Members of *Firmicutes* included *Geobacillus*,* Thermoanaerobacter*,* Thermoanaerobacterium*, Candidatus *Desulforudis*, and *Caldicellulosiruptor* that can participate in different stages of halocellulose degradation as reported in previous studies (Bhalla et al., 2013; Eichorst et al., 2013; De Maayer, Brumm, Mead, & Cowan, 2014; Cobucci‐Ponzano et al., 2015; Vishnivetskaya et al., 2015). Eichorst et al. (2013) suggested that *Firmicutes* are the primary degraders of cellulose in laboratory enrichment experiments. In the decomposition of sugarcane bagasse waste at 50°C, the predominant phylum was *Proteobacteria* (Mhuantong et al., 2015). Thus, these reports elucidated that *Firmicutes* and *Proteobacteria* are the important phyla for biomass degradation at high temperatures. Another important bacterial component in SK‐Y was the *Acidobacteria* phylum, particularly the *Thermoanaerobaculum* (7.3%) genus, a chemo‐organotroph that thrives in anaerobic habitats (Losey et al., 2013). According to Fan et al. (2011), Acidobacteria exclusively or preferentially use organic substrates (in this work, plant litter) as an energy source.

Some of the thermophiles in SK‐Y were also found in other natural geothermal or heated laboratory setups related to biomass degradation (Table 3). Nevertheless, our data suggest that thermophiles involved in biomass degradation under natural conditions are far more complicated than laboratory setups. In one report, only six orders were reported to be involved in an anaerobic digestion of sludge enriched with microcrystalline cellulose at 55°C (Xia et al., 2014), whereas SK‐Y contained more than 100 orders. In separate studies, <10 genera were reported for each case (Table 3). Since these analyses were conducted in a laboratory setup using predetermined nutrients or cultivation conditions (Park et al., 2012; Eichorst et al., 2013; Xia et al., 2014; Yu et al., 2015), the few genera that grow well under these conditions would eventually dominate the culture.

In addition, our data in this work agreed with observations made by Peacock et al. (2013), as microbiota in the SK‐Y water sample differed from the populations in the green biofilm attached to the foliage, as well as the microbiota in the degraded plant litter. During the biomass decomposition process, phyla composition may alter (Eichorst et al., 2013; Yu et al., 2015). The length of time needed for new fallen foliage to reach the degradation stage in SK‐Y remains unknown. However, microorganisms attached to the top layer of the submerged plant litter bed (i.e., submerged foliage with no apparent biofilm and submerged foliage with green biofilm) could mimic taxa that are involved in the early stage of the degradation process. Slowly, over a certain period of time, this foliage is covered and compressed by new layers of fallen plant litter. The initial foliage would eventually be occupied by a slightly different microbiota community to complete the degradation (i.e., the decay sample in this study).

### 4.2 Thermozymes for biomass degradation

To examine the dominant genera in SK‐Y able to degrade biomass, all genera with a relative abundance of ≥0.85% were listed and individually searched against the CAZy complete genome database. The 18 genera with a total of 61 GH families are summarized in Table S2. Genera with incomplete genome data, those with lower optimum growth temperature (<40°C) or OTUs that are unable to be classified confidently to the genus level, or OTUs that account for less than 0.85% of the total population were excluded from the analyses.

Some of the candidates listed in Table S2 have been well characterized, such as enzymes from *Melioribacter* (Podosokorskaya et al., 2013) and *Thermoanaerobacterium* (Currie et al., 2014). The majority of the dominant bacterial genera in SK‐Y produce GH enzymes, supporting the genera listed in Table S2 as being generally important for biomass degradation at circumneutral pH and high temperature. Additionally, we deduced that bacteria, instead of archaea, play a more important role in the consortium that degrades biomass, which is because in most reported articles, bacteria instead of archaea dominated biomass degradation process (Table 3). In addition, genomes of thermophilic archaeal harbor lower numbers of GH groups than thermophilic bacteria (CAZy database). Generally, the genome size of archaea is relatively smaller than bacteria, probably due to the genome streaming process, many genes for GH enzymes in archaea have been omitted (Urbieta et al., 2015).

To date, most well‐studied auxiliary activities (AA) enzymes originate from fungi (Levasseur, Drula, Lombard, Coutinho, & Henrissat, 2013; Karnaouri, Topakas, Antonopoulou, & Christakopoulos, 2014). Based on the complete genome information shown in Table S2, only three AA families (i.e., AA 1 from *Melioribacter roseus* P3M, AA3 from *Meiothermus ruber* DSM 1279, and AA 6 from *Geobacillus stearothermophilus* X1 and *Geobacillus thermoglucosidasius* DSM 2542) were possibly present in SK‐Y. Based on CAZy classification of AA enzymes, genes for AA are lacking or missing from most of the thermophiles listed in Table S2. Several *Thermus* OTUs were identified in SK‐Y as minority taxa. A thermostable and chloride‐tolerant laccase (AA 1, E.C 1.10.3.2) from *Thermus thermophilus* SG0.5JP17‐16 was cloned and overexpressed (Liu et al., 2015). Based on information provided in CAZy, the genome sequence of *T. thermophilus* SG0.5JP17‐16 lacked laccase. This contradictory example suggests that AA list available in the database may require further validation by researchers. In fact, the total number of identified AA enzymes in genome sequencing projects are lower than well‐established GHs as categorized in the CAZy and PeroxiBase Database (Mirete, Morgante, & González‐Pastor, 2016). By further examining the minority genera (<0.85%) detected in SK‐Y, we identified the presence of some AA genes in some sequenced genomes. Examples include *Nocardioides* (AA 3), *Amycolatopsis* (AA 3, AA 6, AA 7, and AA 10), *Azoarcus* (AA 3, AA 4, and AA 6), *Pseudoxanthomonas* (AA 3 and AA 6), and other AA enzymes present in *Anoxybacillus*,* Thermodesulfobacteriaceae*,* Clostridium*,* Geobacter*,* Bacillus*, and some other minority members. Despite being generally mesophilic, proteins from *Bacillus* spp. may be thermostable. For instance, laccase from *Bacillus licheniformis* ATCC 9945a had an optimum temperature of 90°C and a half‐life of 50 min at 70°C (Lončar, Božić, & Vujčić, 2016). We were not able to rule out the presence of fungi or enzymes from fungi in SK‐Y that may assist with partial lignin removal. Nevertheless, as the average temperature of SK‐Y is relatively high, the presence of fungi and the stability of its enzymes are questionable.

## 5 CONCLUSIONS

The work presented here describes the microbiota within a heated biomass degrading bioreactor hot spring. The microbial community within SK‐Y included 25 phyla, 58 classes, 110 orders, 171 families, and 328 genera. Thus, SK‐Y represents a good source for isolation of efficient biomass degrading thermophiles and thermozymes. Biomass degradation at high temperatures may involve various community members. Abiotic factors such as temperature, dissolved oxygen level, and stage of degradation may affect the population of the microbiota members. The microbial profiling data demonstrated that at least 18 genera found in this natural ecosystem are potential candidates for efficient lignocellulosic enzymes. Analysis of these genera, based on existing sequenced genomes, revealed at least 61 GH enzymes that exemplify the important interplay between diverse microorganisms in communities that contribute to the enzyme repertoires required for the degradation of lignocelluloses. Further analyses using metatranscriptomic sequencing may provide more detailed insight into the gene expression level of GHs, and may possibly mine new AA enzymes.

## CONFLICT OF INTEREST

The authors declare that they have no competing interests linked to the data presented in this manuscript. All the authors consented to the publication of this work.
