# Supplementary material for: Microbial diversity of thermophiles with biomass deconstruction potential in a foliage‐rich hot spring
Source: Microbiologyopen. 2018 Mar 30;7(6):e00615. doi: 10.1002/mbo3.615 (PMC6291792; doi:10.1002/mbo3.615)
Supplement: Supplementary file 1 [file MBO3-7-e00615-s001.docx]

**Microbial Diversity of Thermophiles with Biomass Deconstruction Potential in a Foliage-Rich Hot Spring**

**Li Sin Lee^2^, Kian Mau Goh^3^, Chia Sing Chan^3^, Geok Yuan Annie Tan^2^, Wai-Fong Yin^2^, Chun Shiong Chong^3^, Kok-Gan Chan^1,2^***

^1^ Vice Chancellor Office, Jiangsu University, Zhenjiang 212013, PR China

^2^ Institute of Biological Sciences, Faculty of Science, University of Malaya, 50603 Kuala Lumpur, Malaysia

^3^ Faculty of Biosciences and Medical Engineering, Universiti Teknologi Malaysia, 81310 Skudai, Johor, Malaysia

*** Correspondence:**

Kok-Gan Chan^1^

kokgan@um.edu.my

**TABLE S1** Water analysis of the pooled SK-Y water sample

| **Test parameter** | **Method** | **Unit** | **SK-Y** | **SK** | **WHO** |
| --- | --- | --- | --- | --- | --- |
| Physical |  |  |  |  |  |
| Colour ADMI | APHA 2120 F | TCU | 68 | 75 | 15 |
| Turbidity ^d^ | APHA 2130 B | NTU | ND (< 1) | 130 | 5 |
| Chemical |  |  |  |  |  |
| pH | APHA 4500 - H^+^ B | - | 8.6 | 8.2 | 6.5−8.5 |
| Aluminium (Al) | APHA 3111 D | mg/L | ND (< 0.1) | 0.96 | 0.2 |
| Ammonia (N) | APHA 4500 – NH_3_ C / NH_3_ B | mg/L | ND (< 0.1) | ND (< 0.1) | 1.5 |
| Ammonical nitrogen | APHA 4500 – NH_3_ C / NH_3_ B | mg/L | ND (< 0.1) | < 0.2 | - |
| Anionic Detergent (MBAS) ^d^ | APHA 5540 C (Mod.) | mg/L | ND (< 0.5) | ND (< 0.2) | - |
| Arsenic (As) | APHA 3114 B | mg/L | ND (< 0.001) | 0.07 | 0.01 |
| Barium (Ba) | APHA 3111 D | mg/L | ND (< 0.05) | ND (< 0.02) | 0.7 |
| Borate (H_3_BO_3_) ^d^ | APHA 4500 B-C | mg/L | ND (< 1) | - | - |
| Boron (B) | APHA 4500 - B B | mg/L | ND (< 0.10) | 0.06 | 2.4 |
| Bicarbonate (CaCO_3_) ^d^ | APHA 2320 B | mg/L | 97.7 | - | - |
| Biocides (Total) ^d^ | In House | mg/L | ND (< 0.1) | ND | - |
| Bromide (Br^-^) ^d^ | By Ion Chromatography | mg/L | ND (< 1.0) | - | - |
| Cadmium (Cd) | APHA 3111 B | mg/L | ND (< 0.003) | ND (< 0.002) | 0.003 |
| Carbon Chloroform Extract (CCE) ^d^ | In House | mg/L | ND (< 0.5) | ND | 0.3 |
| Calcium (Ca) ^d^ | APHA 3111 B | mg/L | 0.03 | - | - |
| Chloride (Cl^-^) | APHA 4500 - CI^-^ B | mg/L | 0.5 | 2 | 300 |
| Chloroform ^d^ | APHA 6210 B | mg/L | ND (< 0.3) | ND | 0.3 |
| Chromium (Cr III) | In-house Method III ^b^ | mg/L | ND (< 0.01) | - | 0.05 |
| Chromium (Cr VI) | APHA 3500 - Cr B | mg/L | ND (< 0.01) | ND (< 0.02) | 0.05 |
| Copper (Cu) | APHA 3111 B | mg/L | 0.08 | ND (< 0.02) | 2 |
| Cyanide (CN^-^) | In-house Method I ^a^ | mg/L | ND (< 0.01) | ND (< 0.02) | - |
| Fluoride (F^-^) | APHA 4500-F^-^ D | mg/L | 6 | 1.1 | 1.5 |
| Formaldehyde (CH_2_O) | In-House Method V ^c^ | mg/L | ND (< 0.01) | < 0.1 | - |
| Free chloride residual (Cl_2_) | APHA 4500- Cl B | mg/L | 0.3 | < 0.1 | 5 |
| Hardness (CaCO_3_) ^d^ | APHA 2340 B | mg/L | 0.6 | < 1 | 500 |
| Iron (Fe) | APHA 3111 B | mg/L | ND (< 0.05) | 0.65 | 0.3 |
| Lead (Pb) | APHA 3111 B | mg/L | ND (< 0.01) | ND (< 0.02) | 0.01 |
| Magnesium (Mg) ^d^ | APHA 3111 B | mg/L | 0.12 | 0.5 | - |
| Manganese (Mn) | APHA 3111 B | mg/L | ND (< 0.01) | ND (< 0.02) | 0.1 |
| Mercury (Hg) | APHA 3112 B | mg/L | ND (< 0.001) | ND (< 0.001) | 0.006 |
| Mineral oil ^d^ | APHA 5520 F Hydrocarbons | mg/L | ND (< 0.3) | ND (< 0.2) | - |
| Nickel (Ni) | APHA 3111 B | mg/L | ND (< 0.02) | ND (< 0.02) | 0.07 |
| Nitrate nitrogen ^d^ | APHA 4500 - NO^3−^ B | mg/L | 0.29 | < 0.1 | - |
| Nitrite (NO_2_^-^) | Palin Test Kits, Method: AK109 | mg/L | ND (< 0.1) | < 0.1 | 3 |
| Phenol (C_6_H_5_OH) | APHA 5530 B & APHA 5530 D | mg/L | ND (< 0.002) | ND (< 0.002) | - |
| Phosphate (PO_4_^3-^) | APHA 4500 - P C | mg/L | 0.84 | 0.2 | - |
| Potassium (K) ^d^ | APHA 3111 B | mg/L | 2.5 | - | - |
| Selenium (Se) | APHA 3114 B | mg/L | ND (< 0.001) | ND (< 0.005) | 0.04 |
| Silver (Ag) | APHA 3111 B | mg/L | ND (< 0.05) | ND (< 0.02) | - |
| Sodium (Na) ^d^ | APHA 3111 B | mg/L | 34.5 | 27 | 50 |
| Strontium (Sr) ^d^ | APHA 3120 B | mg/L | ND (< 0.6) | - | - |
| Sulfate (SO_4_^2-^) | APHA 4500 - SO_4_^2−^ E | mg/L | 6.4 | 8 | 250 |
| Sulfur (S) ^d^ | Test by ICP-OES | mg/L | 0.5 | 3.9 | - |
| Sulphide | APHA 4500 - S^2-^ F | mg/L | 12.3 | 0.2 | - |
| Total nitrogen | APHA 4500 - N_org_ B | mg/L | ND (< 0.1) | 5.6 | - |
| Zinc (Zn) | APHA 3111 B | mg/L | 0.17 | ND (< 0.02) | 5 |
| Other |  | mg/L |  |  |  |
| Acidity | APHA 2310 B | mg/L | 15.8 | < 1 | - |
| Alkalinity | APHA 2320 B | mg/L | 51.7 | 76 | - |
| Biochemical oxygen demand (BOD) 5 days at 20 °C | APHA 5210 B / APHA 4500 - O G | mg/L | 6 | 5 | - |
| Biochemical oxygen demand (BOD) 5 days at 60 °C | APHA 5210 B / APHA 4500 - O G | mg/L | 4 | 10 | - |
| Biochemical oxygen demand (BOD) 5 days at 80 °C | APHA 5210 B / APHA 4500 - O G | mg/L | 3 | 5 | - |
| Chemical oxygen demand (COD) | APHA 5220 C | mg/L | 17 | 35 | - |
| C:N Ratio (TOC/TN) ^d^ | APHA 5310 D / APHA 4500 - N_org_ B | mg/L | 0.5 | - | - |
| Dissolved oxygen | APHA 4500 - O G | mg/L | 3.0 | 6.2 |  |
| Total organic carbon (TOC) ^d^ | APHA 5310 D | mg/L | 0.502 | 9.04 | - |
| Bacteriological |  |  |  |  |  |
| Total coliform count ^d^ | APHA 9221 B | MPN  (per 100 mL) | ND (< 2) | ND (< 1.1) | ND |
| *Escherichia coli* ^d^ | APHA 9221 F | MPN  (per 100 mL) | ND | ND (< 1.1) | ND |

Abbreviations: SK-Y: Y-shaped Sungai Klah hot spring; SK: Sungai Klah main stream hot spring; WHO: World Health Organization; APHA: Standard Methods of American Public Health Association for the Examination of Water & Wastewater, 21st Edition, 2005; ADMI: American Dye Manufacturer’s Institute; TCU: true color unit; NTU: Nephelometric Turbidity Unit; MPN: most probable number; Mod.: modified method; ND: not detectable

Method reference(s):

^a^ In-house Method 1: Based on APHA 4500 - CN- C & E and Merck Method 14429

^b^ In-house Method III: Based on APHA 3500 - Cr B /APHA 3111 B

^c^ In-house Method V: Based on Macherey Nagel Nanocolor Formaldehyde 8

^d^ Not SAMM Accredited

**TABLE S2** List of important OTUs identified in SK-Y and their representative strains

| **OTU/Genera present in SK-Y hot spring^a^** | **Close match to SK-Y OTU with complete genome information** | **GH^b^** | **References** |
| --- | --- | --- | --- |
| *Acidimicrobium* | *A. ferrooxidans* | 5,13,15,18,23,30,39 | [Clum *et al.* (2009](#_ENREF_2)) |
| *Aeropyrum* | *A. pernix* | 99,122 | [Kawarabayasi *et al.* (1999](#_ENREF_6)) |
| *Caldilinea* | *C. aerophila* | 1,2,3,4,13,16,18,20,23, 29,31,33,36,38,39,42,43,51,63,65,77,78,99,116, 127,130 | n.a |
| *Caldisphaera* | *C. lagunensis* | 15,31,38,57 | n.a |
| *Chloracidobacterium* | *C. thermophilum* | 1,3,13,15,23,27,57,73,77 | [Costas *et al.* (2012](#_ENREF_3)) |
| *Chloroflexus* | *C. aurantiacus*; *C. aggregans* | 1,2,3,5,9,13,15,16,18,23,31,32,35,38,39,51,57,65,77,78,94,114 | n.a. |
| *Desulfurobacterium* | *D. thermolithotrophum* | 23,73 | [Göker *et al.* (2011](#_ENREF_4)) |
| *Fervidobacterium* | *F. islandicum* | 1,3,4,5,13,16,20,23,30,31,35,38,42,57,94,130 | [Lee *et al.* (2015](#_ENREF_8)) |
| *Geobacillus* | *G. thermodenitrificans*; *Geobacillus* sp. | 1,2,3,4,10,13,18,23,32,36,38,39,43,51,52,67,70,73,105,130 | [Wissuwa *et al.* (2016](#_ENREF_12)); [Petkauskaite *et al.* (2017](#_ENREF_9)) |
| *Meiothermus* | *M. silvanus*; *M. ruber* | 1,3,4,10,13,15,23,26,31,36,38,42,43,57,63,77,114,125,130 | [Sikorski *et al.* (2010](#_ENREF_10)); [Thiel *et al.* (2015](#_ENREF_11)) |
| *Melioribacter* | *M. roseus* | 1,2,3,5,9,10,13,16,20,23,26,27,28,29,30,31,35,43,47,50,51,53,55,67,77,88,92,94,97,105,106,115, 125,127,130 | [Kadnikov *et al.* (2013](#_ENREF_5)) |
| *Methanothermococcus* | *M. okinawensis* | 15,57 | n.a |
| *Methanotorris* | *M. igneus* | 15,57 | n.a |
| *Roseiflexus* | *R. castenholzii* | 1,2,3,4,5,13,15,18,20,23,29,36,38,39,51,57,73,77,78,94 | n.a |
| *Thermoanaerobacter* | *T. italicus* | 1,2,3,4,5,10,13,18,23,26,28,31,32,36,38,52,65,67,94,105,130 | n.a. |
| *Thermoanaerobacterium* | *T. saccharolyticum* | 1,2,4,5,10,11,13,15,18,23,25,26,28,29,31,36,39,42,43,51,52,53,65,66,67,84,94,105,120,130 | n.a. |
| *Thermobaculum* | *T. terrenum* | 1,2,3,4,5,10,13,15,29,32,36,38,39,42,43,51 | [Kiss *et al.* (2010](#_ENREF_7)) |
| *Thermosipho* | *T. africanus*; *T. melanesiensis* | 1,2,3,4,13,20,23,31,36, 38,57,73,85,130 | [Antoine *et al.* (1997](#_ENREF_1)) |

Notes:

^a^ OTUs with ≥ 0.85% relative abundance in SK-Y

^b^ Based on http://www.cazy.org


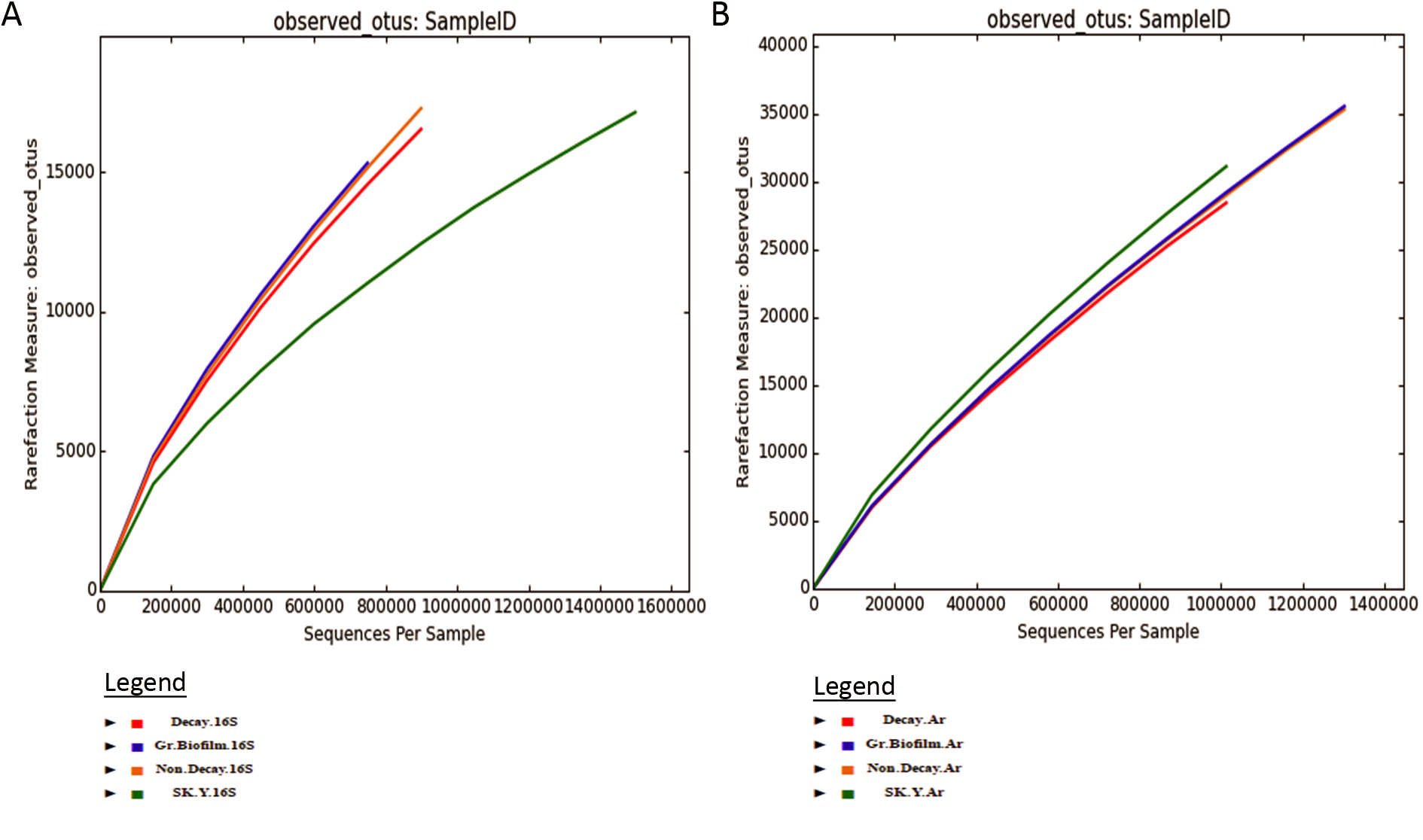
**FIGURE S1** Alpha rarefraction plot based on observed OTUs. (a) Bacterial diversity and (b) archaeal diversity


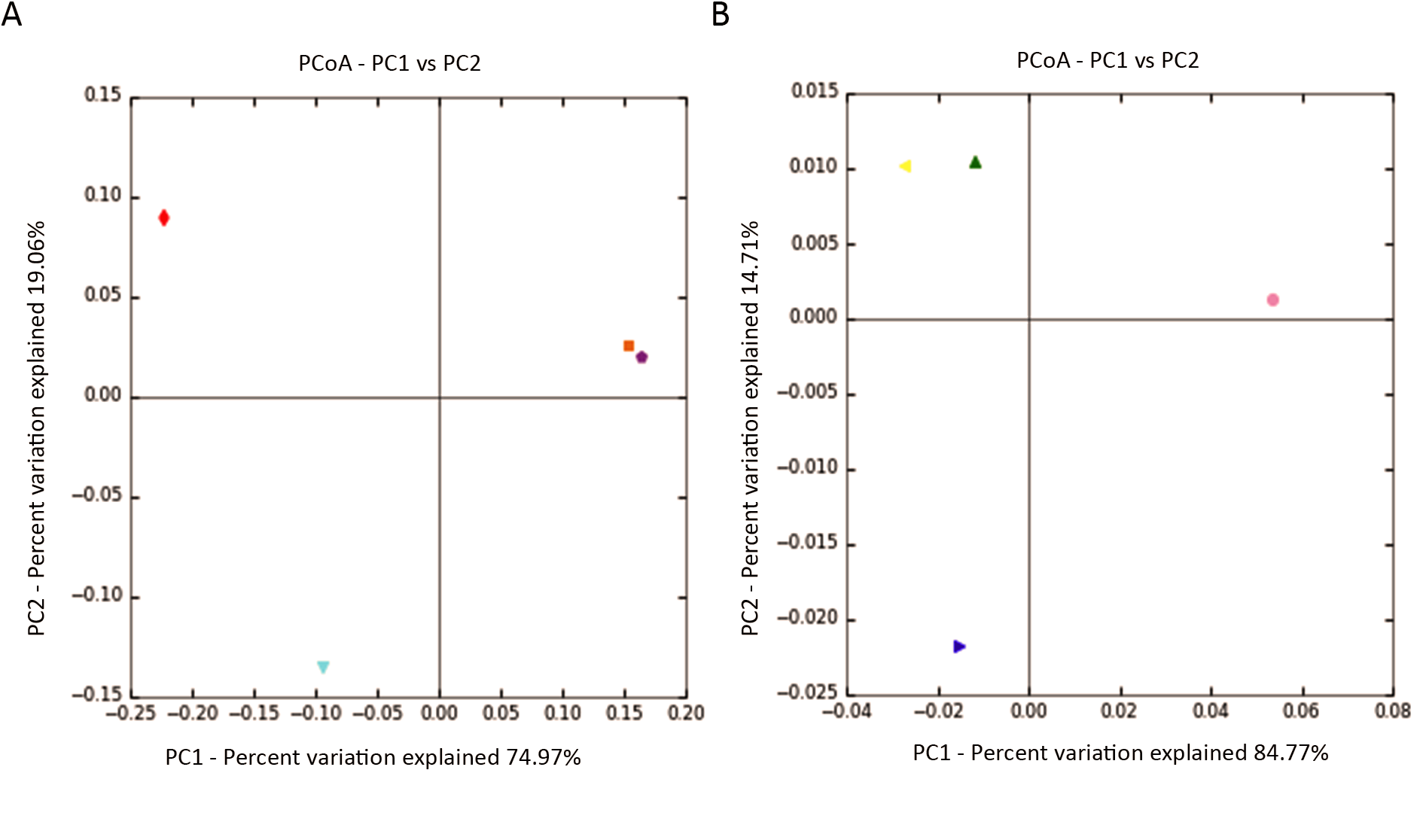
**FIGURE S2** Ordination plots derived from Principal Coordinates Analysis (PCoA) of phylogenetic beta diversity metrics using weighted UniFrac algorithm between bacterial and archaeal community composition of SK-Y. (a) PCoA of bacterial diversity and (b) PCoA of archaeal diversity
